# Seed coat-derived ABA regulates seed dormancy of *Pyrus betulaefolia* by modulating ABA and GA balance

**DOI:** 10.3389/fpls.2025.1667946

**Published:** 2025-09-01

**Authors:** Xiaoting Wang, Lufang He, Jianzhao Li, Songling Bai, Yuanwen Teng, Wei Hui

**Affiliations:** ^1^ Xi’an Botanical Garden of Shaanxi Province, Institute of Botany of Shaanxi Province, Xi’an, China; ^2^ College of Life Sciences, Engineering Research Center for High-Valued Utilization of Fruit Resources in Western China of Ministry of Education, Shaanxi Normal University, Xi’an, China; ^3^ College of Agriculture and Biotechnology, Zhejiang University, Hangzhou, Zhejiang, China; ^4^ School of Horticulture, Ludong University, Yantai, China

**Keywords:** *Pyrus betulaefolia*, seed dormancy, seed coat, ABA, GA, ABI5, GA2ox

## Abstract

Plant seeds have evolved diverse dormancy types and regulatory mechanisms to adapt to environmental conditions and seasonal changes. As a commonly used rootstock for cultivated pears, *Pyrus betulaefolia* faces challenges in seedling production and large-scale cultivation due to limited understanding of seed dormancy mechanisms. In this study, we report that *Pyrus betulaefolia* seeds exhibit non-deep physiological dormancy, with seed coats playing a pivotal regulatory role. Exogenous abscisic acid (ABA) treatment, fluridone application and seed coat bedding assay demonstrated that dormant seed coats actively synthesized ABA to inhibit embryo germination during imbibition. ABA in imbibed dormant seed coats stimulated ABA biosynthesis in embryos, leading to increased expression of genes involved in ABA biosynthesis (*PbeNCED-3*) and ABA-responsive (*PbeABI3-1*, *PbeABI5-1*, and *PbeABI5-5*). Importantly, PbeABI5-5 directly binds to the promoters of *GIBBERELLIN 2-OXIDASE 3/4* (*PbeGA2ox-3/4*) to activate their transcription. We establish that in dormant *Pyrus betulaefolia* seeds, the seed coat controls embryo dormancy release through coordinated regulation of PbeABI5-GA2ox module, thereby maintaining the critical balance between ABA and GA.

## 1 Introduction

*Pyrus betulaefolia* is a forest tree species that belongs to *Pyrus* (Rosaceae). It exhibits remarkable environmental adaptability and thrives under various abiotic stresses, including drought, freezing temperatures, and waterlogged conditions. In horticultural cultivation, it is primarily used as a rootstock for pear trees and serves as a crucial parental material in breeding dwarfing rootstocks and disease-resistant pear varieties (Li et al., 2017; Thakur et al., 2024). It is also an exceptionally valuable germplasm resource for fruit trees (Li et al., 2015). *Pyrus betulaefolia* is seed-propagated but exhibits physiological dormancy. Seed dormancy represents a crucial adaptive strategy in plants, ensuring viable seeds remain temporarily incapable of germination period even when exposed to favorable environmental conditions such as optimal temperature, moisture, and light (Kim et al., 2024; Baskin and Baskin, 2007). This intrinsic biological mechanism, distinct from quiescence where seeds merely await suitable external conditions (Shu et al., 2016a), plays a fundamental role in plant life history strategies and ecological success. Cold stratification, the conventional dormancy-breaking technique, is constrained by mycotic susceptibility and viability decline during suboptimal treatment. Elucidating the mechanisms underlying seed dormancy—including its physiological basis and molecular regulation is critical for advancing propagation techniques. Such research will not only enhance seedling production efficiency but also support breeding initiatives aimed at improving pear rootstocks and cultivars (Bao and Zhang, 2010). Therefore, this study seeks to investigate the dormancy characteristics of *Pyrus betulaefolia* seeds, with the ultimate goal of developing optimized germination protocols for commercial and conservation applications.

Abscisic acid (ABA) serves as the primary germination inhibitor in seeds (Barrero et al., 2010). ABA is actively synthesized in seed coats and subsequently transported to embryos, where it effectively suppresses germination and maintains dormancy (Lee et al., 2010). Therefore, while the testa (outer seed coat) primarily imposes physical constraints, the endosperm (inner nutritive tissue) functions as a dynamic biochemical barrier that synthesizes and releases ABA to suppress germination. In *Arabidopsis thaliana*, seed coat removal triggers the plant embryo growth and greening (Debeaujon et al., 2000). ABA can inhibit the pre-harvest sprouting phenotype of seeds, and ABA content increases with the onset of dormancy (Aloryi et al., 2025). In the ABA signaling pathway, 9-cis-epoxycarotenoid dioxygenases (NCED) serve as the rate-limiting enzyme for ABA biosynthesis, which regulates ABA levels by catalyzing carotenoid cleavage. In Arabidopsis, triple mutant *nced5/nced6/nced9* greatly reduced seed dormancy compared with *aba2-2* (Frey et al., 2012). ABA-insensitive (ABI) transcription factors constitute core components of ABA signal transduction. Many allelic mutations of *abi3* have been found in *Arabidopsis thaliana*, with the strongest phenotype being *abi3-5*, and the mutant seeds remain green and are not sensitive to ABA during germination (Ooms et al., 1993). ABI5, an ABA-responsive element-binding factor (AREB/ABF) that binds to ABA response elements (ABREs) in the promoters of ABA-regulated genes, negatively regulates seed germination (Shu et al., 2016b).

Gibberellins (GAs) represent another crucial class of phytohormones that play pivotal roles in breaking seed dormancy and promoting germination, primarily through their antagonistic interaction with ABA (Shu et al., 2018). During seed maturation, GA biosynthesis is actively suppressed, resulting in remarkably low GA levels in mature quiescent seeds (Jacobsen et al., 2002). However, upon imbibition, the scutellum epithelial cells of the embryo rapidly activate GA biosynthesis, leading to a significant accumulation of bioactive GAs in both the embryo and endosperm (Henderson et al., 2004). Gibberellin biosynthesis during seed germination is primarily regulated by GA20ox and GA3ox, which catalyze the production of bioactive GAs, while GA2ox plays a key role in GA deactivation to maintain dormancy (Hedden, 2020). GA burst triggers the production of various hydrolases, including α-amylase, which mobilize stored reserves in the endosperm by converting them into carbohydrates and other nutrients essential for germination (Hedden, 2025). It is the dynamic balance between ABA and GA, rather than their absolute concentrations, that serves as the critical determinant for seed dormancy and germination (Graeber et al., 2012). This balance is maintained through complex reciprocal regulation of hormone biosynthesis and catabolism. ABI4 can directly target the promoters of ABA metabolic genes *CYP707A1* and *CYP707A2* to promote ABA accumulation and impair GA accumulation during seed dormancy (Shu et al., 2013). Similarly, CHO1 (CHOTTO1), also regulates seed dormancy, but it plays a regulatory role in upstream of ABI4 (Yamagishi et al., 2009; Yano et al., 2009). Cantoro et al. found that SbABI4 and SbABI5 can directly bind to the promoter of *SbGA2ox3 in vitro*, which may activate GA degradation, thus preventing germination of dormant grains (Cantoro et al., 2013). However, further evidence is needed to confirm the occurrence of this interaction *in vivo*. Additionally, the GA signaling pathway is initiated when gibberellin binds to its receptor, GIBBERELLIN INSENSITIVE DWARF 1 (GID1), forming the GA-GID1-DELLA complex (Sun, 2011). This complex recruits the SCFSLY1/GID2 E3 ubiquitin ligase, leading to the degradation of DELLA proteins - a critical step in activating downstream GA-responsive genes (Wang and Deng, 2014). In Arabidopsis, five DELLA proteins have been identified: GA-INSENSITIVE (GAI), REPRESSOR OF *ga1-3* (RGA), and three RGA-LIKE proteins (RGL1, RGL2, RGL3) (Bao et al., 2020). Among these, RGL2 plays a specialized role by forming a transcriptional complex with ABI4, which mediates the antagonistic interaction between ABA and GA, precisely controlling the shift from dormancy to germination (Liu et al., 2016). Under low GA conditions, DELLA proteins remain stable, exerting a dual regulatory function: they not only suppress GA signaling by modulating downstream targets but also inhibit the expression of key GA biosynthesis (*GA20ox2*, *GA3ox1*) and signaling (*GID1*) genes, thereby maintaining seed dormancy (Kim et al., 2023).

Despite evidence implicating seed coats in *Pyrus betulaefolia* dormancy, precise classification of its dormancy type and underlying regulatory mechanisms remains unresolved. In this study, we systematically investigated the role of seed coats in maintaining embryo dormancy in *Pyrus betulaefolia*. Our findings demonstrated that the seeds exhibit non-deep physiological dormancy. Through seed coat bedding assays, we identified that the seed coats of dormant *Pyrus betulaefolia* significantly suppress embryo germination while simultaneously upregulating key dormancy-related genes, including *PbeNCED-3*, *PbeABI3-1*, *PbeABI5-1*, *PbeABI5-5*, *PbeGA2ox-3*, and *PbeGA2ox-4* in the embryo. Molecular and biochemical analyses further revealed that transcription factor PbeABI5-5 plays a pivotal role in dormancy maintenance by directly binding to the promoters of *PbeGA2ox-3* and *PbeGA2ox-4* to activate their transcription. These results provide compelling evidence that PbeABI5-GA2ox module serves as a central regulator of ABA/GA balance in *Pyrus betulaefolia*, offering new insights into the molecular basis of seed dormancy in this economically important species.

## 2 Results

### 2.1 Seed dormancy in *Pyrus betulaefolia* is imposed by seed coat

**Figure 1:**
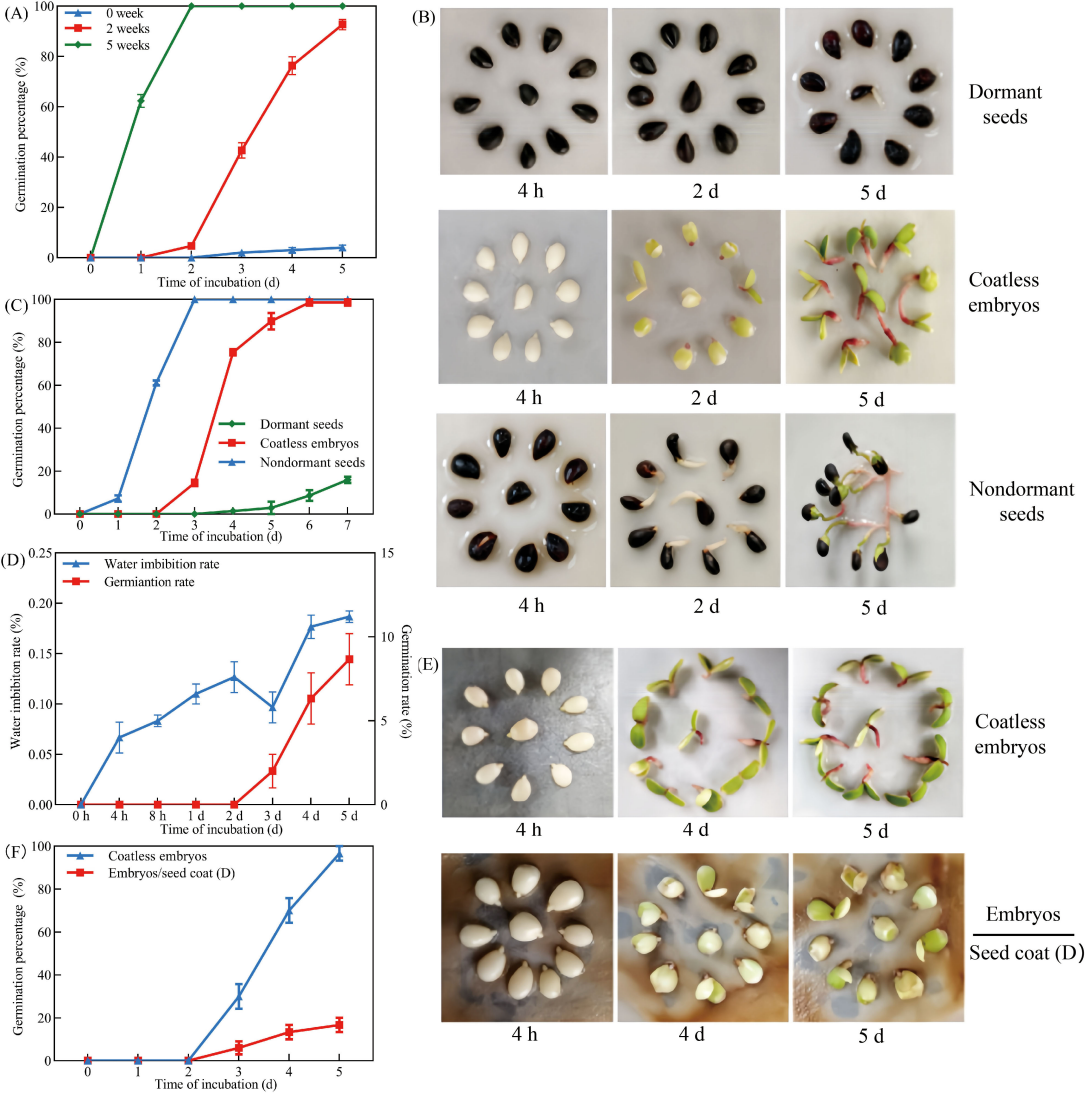
The dormant seed coats repressed germination of *Pyrus betulaefolia* embryos. **(A)** Percentage of seed germination after 0, 2 and 5 weeks of cold stratification. **(B)** Representative images of seed germination assays of untreated (Dormant seeds), coatless embryos dissected from dormant seeds (Coatless embryos), after 5 weeks of cold stratification (Nondormant seeds). **(C)** Quantification of the germination frequencies of seeds as shown in **(B, D)** The water imbibition rate and germination rate of dormant seeds. **(E)** Seed coats bedding assay using embryos dissected from dormant seeds and seed coats dissected from dormant seeds. Seed coats and coatless embryos were physically separated by filter paper. Pictures were taken 4 h, 4 and 5 d after imbibition. **(F)** Quantification of the germination frequencies of seeds as shown in **(E)**. All data represent the mean ± SE of three biological replicates (> 30 seeds per replicate). Similar results were obtained from three different seed lots.

Cold stratification was used to examine the dormancy-breaking capacity of freshly harvested *Pyrus betulaefolia* seeds. Non-stratified seeds demonstrated strong dormancy (hereinafter referred to as “dormant seeds”) (**Figure 1A**). In contrast, seeds subjected to 2-week cold stratification showed significantly enhanced germination potential, reaching 93.33% after 5 days of incubation at 21°C. Maximal dormancy release was achieved following 5 weeks of cold stratification, resulting in 100% germination (hereinafter referred to as “nondormant seeds”), suggesting the experimental seeds possessed dormancy while maintaining high viability (**Figure 1A**). To elucidate the mechanisms underlying seed dormancy in *Pyrus betulaefolia*, germination assays were conducted using isolated embryos dissected from dormant seeds (hereinafter referred to as “coatless embryos”). Comparative analysis revealed that while excised embryos from dormant seeds exhibited delayed germination compared to non-dormant seeds, they ultimately achieved 100% germination within 6 days of incubation (**Figures 1B, C**). In stark contrast, intact dormant seeds showed a germination rate of 8.70%. These findings provided compelling evidence that the seed coat served as the primary constraint inhibiting germination in *Pyrus betulaefolia* seeds.

Seed coat-imposed dormancy in seeds involves three potential mechanisms, including physical impermeability to water, mechanical constraint, and endogenous inhibitors. To investigate the role of water impermeability, we measured water uptake kinetics in dormant seeds. As defined by Baskin and Baskin (2007), the seeds we used exhibited triphasic water uptake kinetics (Phase I: rapid 0–8 h absorption; Phase II: 8 h-3 d plateau; Phase III: post-3 d surge coupled with germination), confirming unimpeded water penetration through the seed coat (**Figure 1D**). The synchronized onset of Phase III and radicle emergence further verified that dormancy arrest occurs post-hydration, implicating physiological constraints. Using a seed coat bedding assay to physically separate seed coats from embryos (**Figures 1E, F**), germination rate of isolated embryos was 100% efficiency, while those cultured with dormant seed coats showed significantly suppressed germination rate (13.33% after 5 days of incubation). Together, these results demonstrated that dormancy of *Pyrus betulaefolia* seed was primarily mediated by inhibitors within the seed coats, which are gradually eliminated or degraded during cold stratification.

### 2.2 Endogenous ABA in dormant seed coats suppresses embryo germination in *Pyrus betulaefolia*

**Figure 2:**
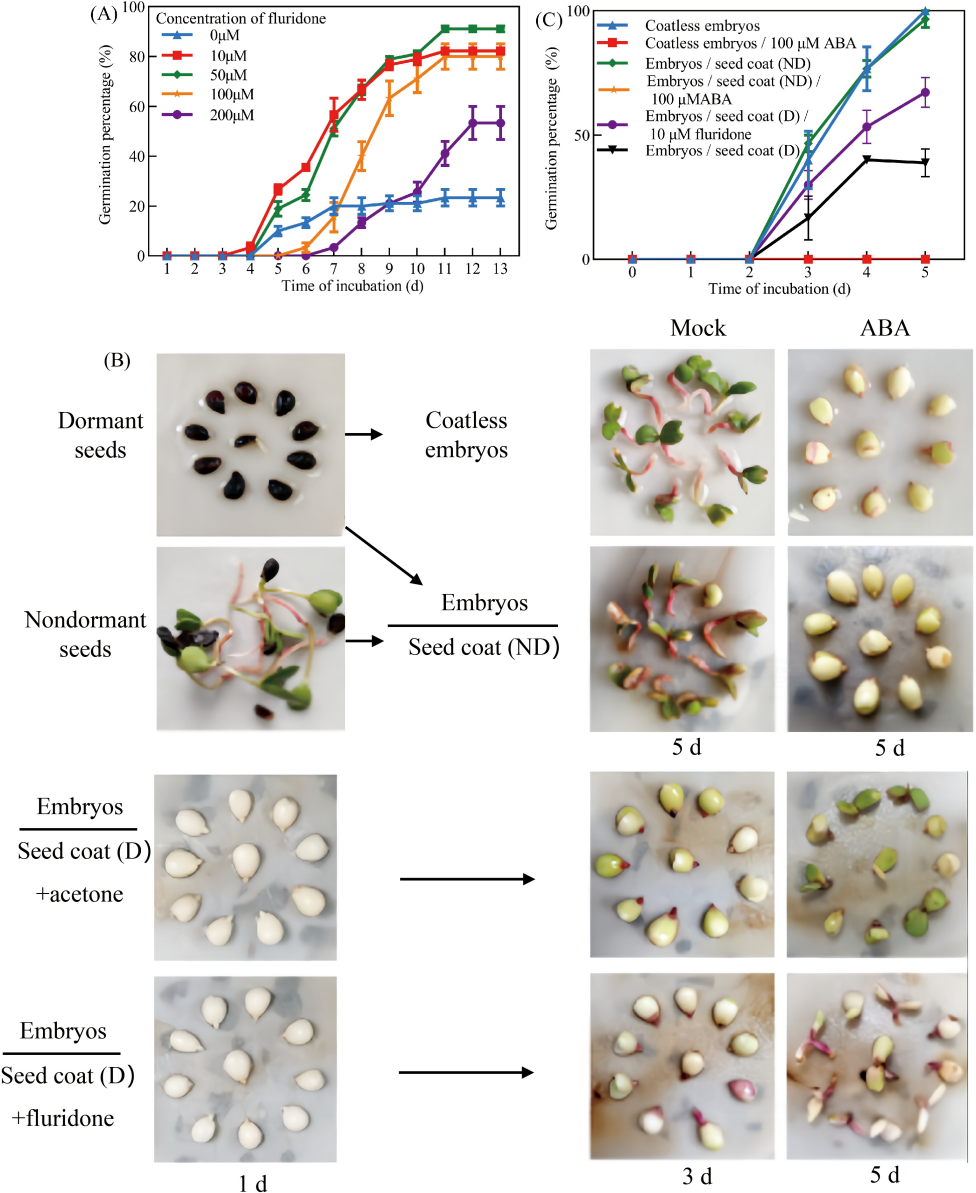
ABA repressed the germination of *Pyrus betulaefolia* embryos. **(A)** Germination percentage of dormant seed in the presence of various concentrations of exogenous fluridone. **(B)** Seed coats bedding assay in the absence or presence of 100 μM ABA using embryos dissected from dormant seeds and seed coats dissected from non-dormant seeds, and seed coats bedding assay in the absence or presence of 10 μM fluridone using embryos dissected from dormant seeds and seed coats dissected from dormant seeds, seed coats and coatless embryos were physically separated by filter paper. **(C)** Quantification of the germination frequencies of embryos as is shown in **(B)**. All data represent the mean ± SE of three biological replicates (> 30 seeds per replicate). Similar results were obtained from three different seed lots.

Given the well-established role of ABA in promoting seed dormancy and suppressing germination, the effects of fluridone, an ABA biosynthesis inhibitor, on dormant seeds in *Pyrus betulaefolia* were investigated. Treatment with 10 μM fluridone significantly promoted germination, achieving an 82.22% germination rate after 13 days under 16h/8h (light/dark) photoperiod at 22 ± 2°C (**Figures 2A, B**). To further examine the role of ABA in seed coat-mediated dormancy,seed coat bedding assays were conducted with or without 100 μM ABA supplementation (**Figure 2C**). Isolated embryos exhibited 100% germination, and non-dormant seed coats had no inhibitory effect. However, ABA completely suppressed germination in both isolated embryos and embryos co-cultured with non-dormant seed coats (**Figure 2C**), suggesting that dormant seed coats function similarly to exogenous ABA in preventing embryo germination. Besides, application of 10 μM fluridone in the seed coat bedding assay significantly attenuated the inhibitory effect of dormant seed coats, restoring embryo germination capacity (**Figure 2C**). These findings provide compelling evidence that dormant seed coats inhibit germination through endogenous ABA accumulation, and that this inhibition can be reversed by blocking ABA biosynthesis.

### 2.3 Dormant seed coats regulate ABA biosynthesis and signaling in *Pyrus betulaefolia* embryos

**Figure 3:**
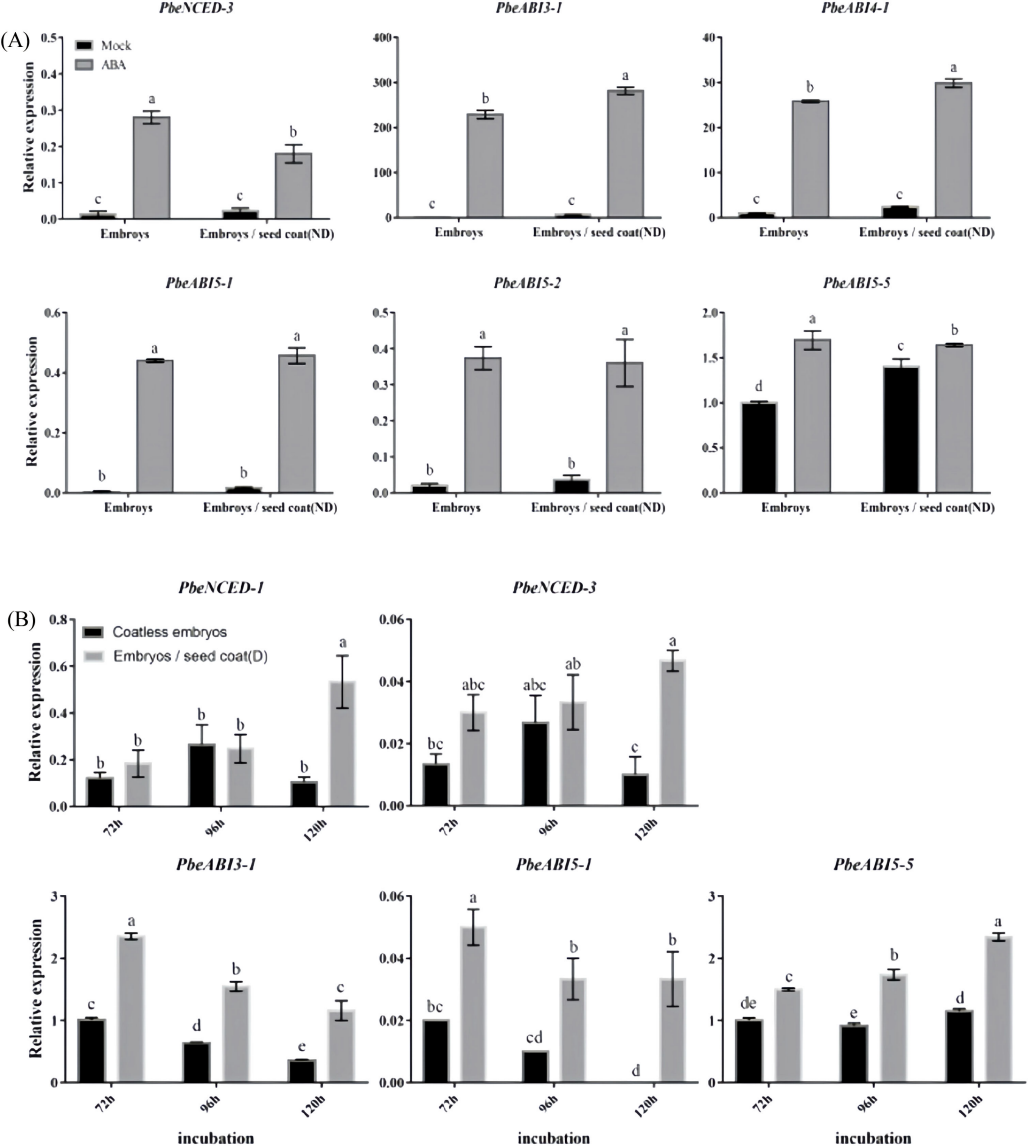
Expression profiles of ABA related genes at different conditions. **(A)** Total RNA was extracted from the coatless embryos and the embryos co-incubated with nondormant seed coats sampled after 120 h of incubation in the absence or presence of 100 μM ABA. **(B)** Total RNA was extracted from the coatless embryos and the embryos co-incubated with dormant seed coats sampled at 72, 96, and 120 h after beginning of incubation. Relative expression levels of ABA-related genes were determined by qRT-PCR as described in the Materials and Methods. All data represent the mean ± SE of three biological replicates (> 30 seeds per replicate). Similar results were obtained from three different seed lots. For all panels, different letters indicate significant differences between the data (*p* < 0.05) using one-way ANOVA with Dunnett’s test.

Expression patterns of key ABA metabolic and signaling genes in coatless embryos treated with either exogenous ABA or dormant seed coats were analyzed (**Figure 3**; **Supplementary Figure 1**). Exogenous ABA treatment significantly increased transcript levels of the ABA biosynthesis gene *PbeNCED-3* in both isolated embryos and embryos co-cultured with nondormant seed coats (**Figure 3A**). Similarly, dormant seed coats markedly enhanced the expression of ABA biosynthesis genes *PbeNCED-1* and *PbeNCED-3* (**Figure 3B**). Furthermore, we observed that exogenous ABA upregulated ABA signaling transcription factors *PbeABI3-1*, *PbeABI4-1*, *PbeABI5-1*, *PbeABI5-2*, and *PbeABI5-5* (**Figure 3A**), while dormant seed coats specifically induced *PbeABI3-1*, *PbeABI5-1* and *PbeABI5-5* expression (**Figure 3B**). These transcriptional profiles implied that dormant seed coats modulated ABA homeostasis through coordinated regulation of *NCED* genes and selective activation of ABI3/ABI5-family signaling components, thereby maintaining seed dormancy.

### 2.4 Seed coat-enforced dormancy involves ABA-mediated GA metabolism

**Figure 4:**
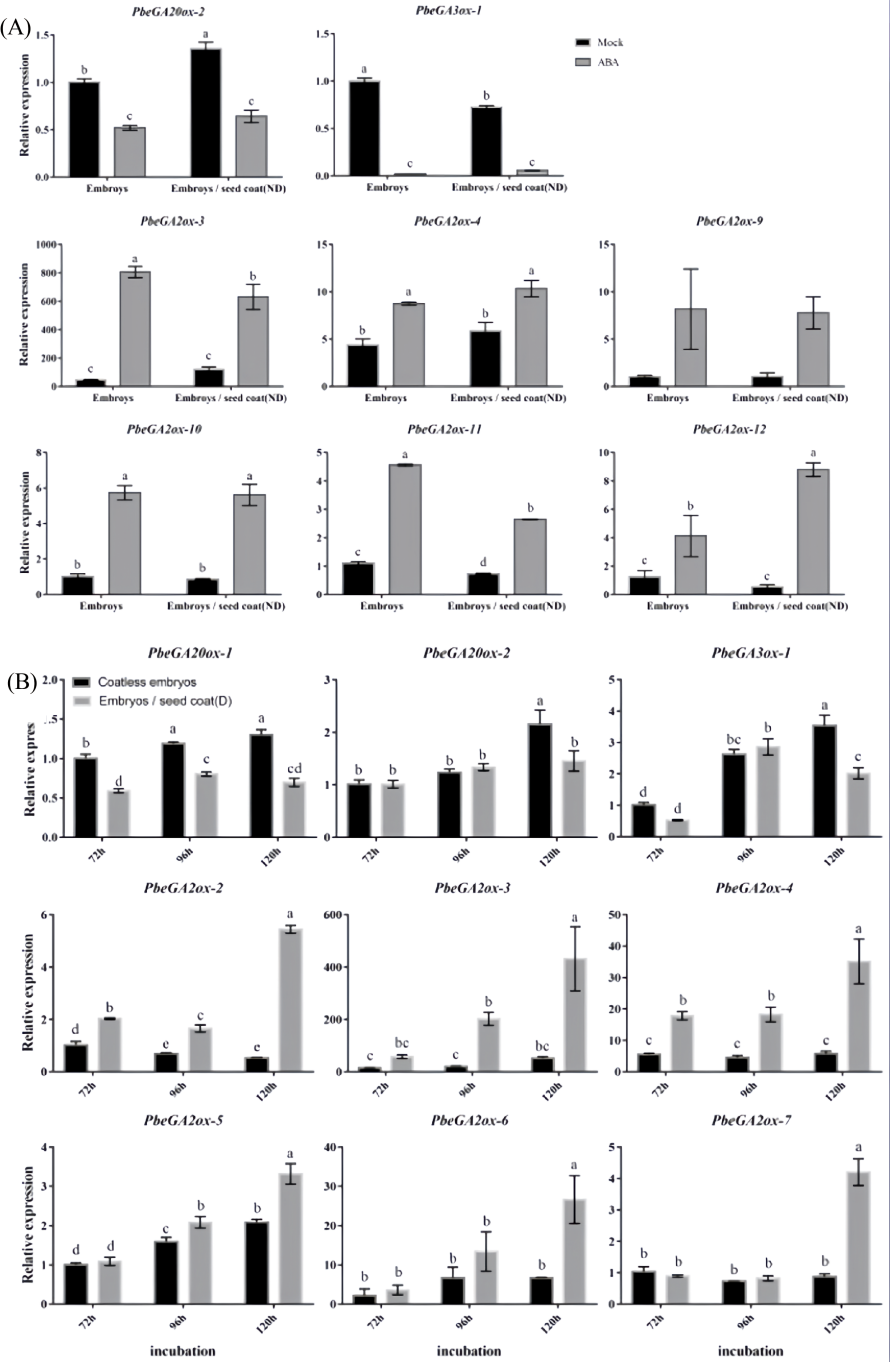
Expression profiles of GA related genes at different conditions. **(A)** Total RNA was extracted from the coatless embryos and the embryos co-incubated with nondormant seed coats sampled after 120 h of incubation in the absence or presence of 100 μM ABA. **(B)** Total RNA was extracted from the coatless embryos and the embryos co-incubated with dormant seed coats sampled at 72, 96, and 120 h after beginning of incubation. Relative expression levels of GA-related genes were determined by qRT-PCR as described in the Materials and Methods. All data represent the mean ± SE of three biological replicates (> 30 seeds per replicate). Similar results were obtained from three different seed lots. For all panels, different letters indicate significant differences between the data (*p* < 0.05) using one-way ANOVA with Dunnett’s test.

**Figure 5:**
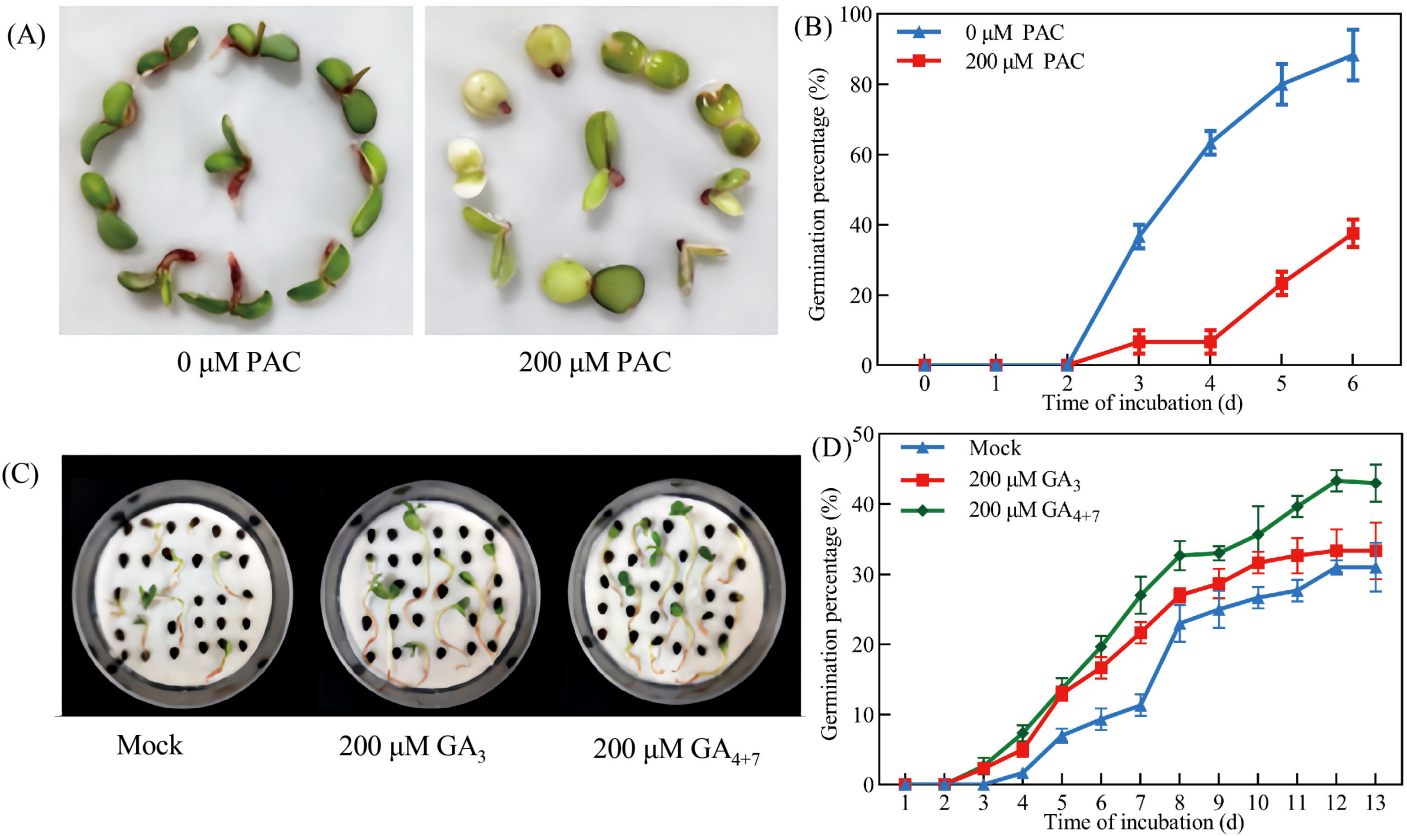
GA increased the germination of *Pyrus betulaefolia* seeds. **(A)** Effect of PAC on the germination of dormant seeds. Pictures were taken 6 d after imbibition. **(B)** Germination percentage of dormant seed in the absence or presence of 200 μM PAC. **(C)** Effect of GA on the germination of dormant seeds. Pictures were taken 13 days after imbibition. **(D)** Germination percentage of dormant seed in the absence or presence of 100 μM GA_3_ or GA_4 + 7_. The bars represent the mean ± SEM of three biological replicates. All data represent the mean ± SE of three biological replicates (> 30 seeds per replicate). Similar results were obtained from three different seed lots.

Investigation of GA metabolic gene expression revealed that both exogenous ABA treatment and dormant seed coats significantly downregulated GA biosynthetic genes (*PbeGA20ox-1*, *PbeGA20ox-2*, and *PbeGA3ox-1*) while concurrently upregulating GA catabolic genes (*PbeGA2ox-2* to *PbeGA2ox-4*, *PbeGA2ox-6*, and *PbeGA2ox-9* to *PbeGA3ox-12*) in coatless embryos, with *PbeGA2ox-3* and *PbeGA2ox-4* showing strong induction (4-18-fold and 2-6-fold increases, respectively) under both treatments (**Figure 4**). Notably, qRT-PCR analysis demonstrated stable expression of DELLA protein family genes (*PbeGAI-Like-1* to *PbeGAI-Like-3* and *PbeRGL1-Like-1* to *PbeRGL1-Like-3*) regardless of treatment (**Supplementary Figure 2**). Additionally, coatless embryos were highly sensitive to the GA biosynthesis inhibitor PAC, while exogenous GA_4 + 7_ could partially overcome dormancy, though both treatments exhibited similar early germination patterns that exceeded controls (**Figure 5**). These findings demonstrated that seed coat-imposed dormancy involved ABA-mediated suppression of GA biosynthesis coupled with enhanced GA catabolism.

### 2.5 PbeABI5 directly binds to the *PbeGA2ox* promoters *in vivo*

**Figure 6:**
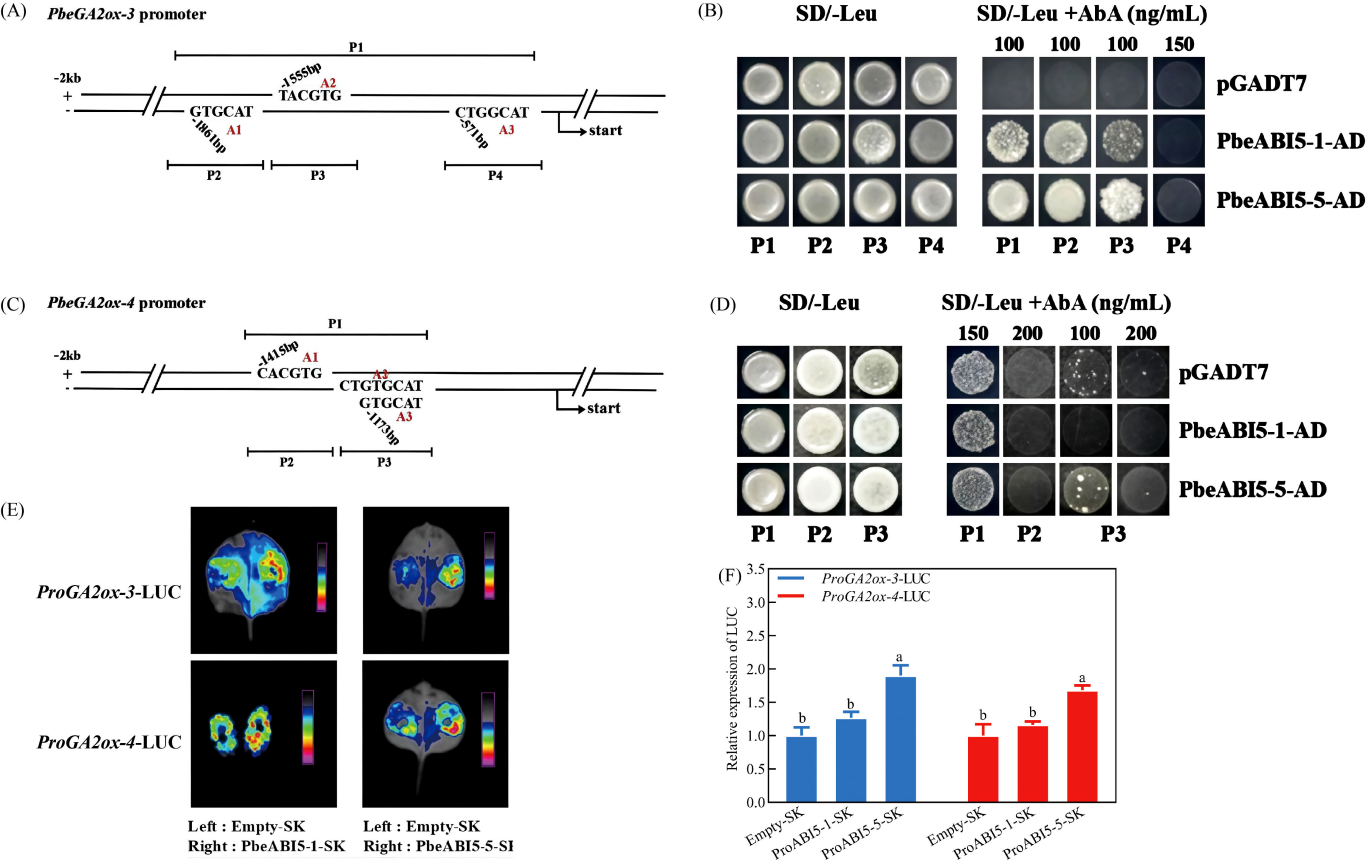
PbeABI5-5 directly bound to the promoter regions of the *PbeGA2ox-3* and *PbeGA2ox-4* to activate their expression. **(A-D)** The *PbeGA2ox-3* gene promoter was divided into P1 fragment (containing A1, A2, and A3 sites, 1368 bp), and P2 fragment (containing A1 site, 202 bp), P3 fragment (containing A2 site, 252 bp), and P4 fragment (containing A3 site, 309 bp); and the promoter region of *PbeGA2ox-4* was divided into P1 segment (containing A1, A2, and A3 sites, 341 bp), and P2 fragment (containing A1 site, 116 bp), P3 fragment (containing A2 and A3 site, 64 bp). The linearized constructs containing different promoter fragments (P1, P2, P3 or P4) in pAbAi were integrated into the genome of Y1HGold yeast strain and either PbeABI5-1-AD, PbeABI5-5-AD or empty AD vector were introduced into each. Aureobasidin A (AbA), an inhibitor of yeast cell growth, was used as a screening marker. Yeast growth on the SD/-Leu+AbA medium indicated binding. **(E, F)** The effects of PbeABI5-1/5 on the promoter activity of *PbeGA2ox-3* and *PbeGA2ox-4* in the luciferase reporter assay. These results are obtained through the NightSHADE LB 985 imaging system and Dual-Luciferase^®^ Reporter Assay System. The experiment was performed independently six times with six leaves infiltrated with each plasmid. The bars represent the mean ± SEM of six biological replicates. Different letters indicate a significant difference among different groups (*p* < 0.05) using one-way ANOVA with Dunnett’s test.

Based on the observed co-expression patterns of *PbeABI5-1*, *PbeABI5-5*, *PbeGA2ox-3* and *PbeGA2ox-4* during seed germination, the potential transcriptional regulation of *GA2ox* genes by ABI5 transcription factors was investigated. Bioinformatics analysis identified three ABRE motifs in the 2-kb promoter regions of both *PbeGA2ox-3* and *PbeGA2ox-4* (**Figure 6A**). Yeast one-hybrid assays revealed that PbeABI5-1 and PbeABI5-5 bound to multiple regions of the *PbeGA2ox-3* promoter, while only PbeABI5-5 interacted with the *PbeGA2ox-4* promoter (**Figures 6B, D**). Transient expression assays in *Nicotiana benthamiana* leaves demonstrated that PbeABI5-5 significantly activated transcription from both the *PbeGA2ox-3* and *PbeGA2ox-4* promoters, as shown by both qualitative and quantitative LUC reporter assays (**Figures 6E, F**). These findings establish that PbeABI5-5 directly binds to and activates transcription of GA catabolic genes, providing a molecular mechanism for the coordinated regulation of ABA signaling and GA metabolism during seed germination.

## 3 Discussion

### 3.1 *Pyrus betulaefolia* seeds exhibit non-deep physiological dormancy with seed coat as ABA source

Seeds of *Pyrus betulaefolia* have a dormant stage, in which embryos with morphological dormancy are not yet fully developed, and they usually germinate after being stored for a period of time at room temperature (Zhang et al., 2022). Our study shows that the germination rate of *Pyrus betulaefolia* dormant seeds is low, and even if stored at room temperature for a period of time, the germination rate did not increase (**Figure 1**). Considering that the germination rate of coatless embryos reached 100% after 6 days of incubation, together with rapid germination of coatless embryos, fully developed embryos and permeable seed coats, we confirmed that *Pyrus betulaefolia* seeds exhibit physiological dormancy. Following Baskin’s classification (Baskin and Baskin, 2007), we identified non-deep physiological dormancy based on three key traits: (1) coatless embryos germinated like non-dormant seeds (**Figure 1**), (2) cold stratification (5 weeks) fully released dormancy (**Figure 1**), and (3) GA_4 + 7_ treatment significantly enhanced germination (**Figure 5**). Subsequent investigations could focus on performing detailed comparative analyses of dormancy depth throughout the Rosaceae family, with particular emphasis on identifying phylogenetic patterns and ecological correlates that may explain the evolutionary development and maintenance of these dormancy strategies.

In the mature seed of most angiosperms, the embryo is encased by two covering layers (coats): the living endosperm tissue and the outer layer of dead tissue (Topham et al., 2017). Experiments involving the removal of external testa layer while leaving the endosperm layer have shown that the endosperm provides most if not all of the germination repressive activity (Bethke et al., 2007). Previous reports have shown that seed coats surrounding the embryo contain compounds possessing germination-inhibitory activities, including ABA (Shimizu et al., 2022). Indeed, gene expression studies in isolated Arabidopsis seed coat tissue have shown that the endosperm expresses genes involved in ABA metabolism, and it has been demonstrated that the seed coat contains ABA (Barrero et al., 2010). While our data confirmed physiological dormancy in *Pyrus betulaefolia* (**Figure 1** and **Figure 2**), the role of seed coat extends beyond a passive barrier. Crucially, dormant seed coats actively synthesize ABA to maintain dormancy, as demonstrated in Arabidopsis by Lee et al. (2010), seed coat bedding assay reveals that imbibed dormant coats release ABA to suppress even non-dormant embryos. This mechanism seems conserved across species - in capeweed (*Arctotheca calendula*), seed coat-derived ABA imposes shallow dormancy (Chapman, 2000), while in recalcitrant *Avicennia marina* seeds, the pericarp (homologous to seed coats) retains high ABA levels that constrain germination post-dehydration (Farrant et al., 1993).

The germination rate was significantly higher than that of the control group when the seeds and embryos were treated with fluridone (**Figure 2**). Consistently, in Arabidopsis, treating imbibed dormant wild-type seeds with fluridone will trigger their germination (Debeaujon and Koornneef, 2020; Millar et al., 2006), suggesting that repression of germination is an active process requiring *de novo* and constitutive production of the phytohormone ABA upon seed imbibition. Further supported by the observation that dormant and nondormant dry seeds contain similarly high amounts of ABA, which played an essential and well established developmental role during seed maturation, and upon seed imbibition, the high ABA levels present in both dormant and nondormant seeds drop drastically by about 10-fold within the first 12 h, and what indeed distinguishes dormant seeds from nondormant seeds is the capacity of dormant seeds to synthesize ABA *de novo* and constitutively once the initial drop of ABA levels has occurred upon seed imbibition (Ali-Rachedi et al., 2004; Piskurewicz et al., 2008), indicating that *de novo* ABA produced in the *Pyrus betulaefolia* seed coats and released toward the embryo to repress germination. Furthermore, the reported observations should not be meant to imply that the ABA derived from the seed coats is the only cause of dormancy. The strength of dormancy in a given seed, i.e., its capacity to restrain from germinating upon imbibition, is most likely related to the seed’s capacity to synthesize ABA as a whole: in both the endosperm and the embryo. Rather, we wish to propose that the endosperm is an essential contributor to sustain dormancy, i.e., to prevent germination over time upon imbibition.

### 3.2 ABA biosynthesis and catabolic feedback shape dormancy maintenance

That ABA synthesized in embryos also contributes to overall seed dormancy is most likely reflected by the observation that dormant embryos green and germinate slower than nondormant embryos upon dissection, and coatless embryos dissected from isolated dormant seeds 24 h after imbibition contained 2.5-fold more ABA than coatless embryos isolated from nondormant seeds (Lee et al., 2010). In agreement with this view, we found that the dormant seed coats promoted the expression of the ABA biosynthesis genes *PbeNCED-1* and *PbeNCED-3* in embryos (**Figure 3**). It should be pointed out that dormancy-promoting function of the seed coat is not strictly restricted to the endosperm. However, *PbeABI3-1* was significantly up-regulated under exogenous ABA or dormant seed coat treatment, which was similar to the expression patterns of *PbeABI5-1* or *PbeABI5-5* (**Figure 3**, **4**). Studies have also shown that ABI3 has a domain interacting with ABI5, and ABI3 can also positively regulate the expression of *ABI5* (Lopez-Molina et al., 2001; Nakamura et al., 2001; Lopez-Molina et al., 2002), indicating the relationship between PbeABI3 and PbeABI5 need to be further studied. Even with a larger relative increase, the absolute amount of *PbeABI5-1* transcript induced by exogenous ABA or dormant seed coat treatment might still be considerably less than the basal and induced levels of *PbeABI5-5* (**Figure 3**, **4**). Crucially, our functional assays clearly demonstrated that only the PbeABI5-5 protein possesses the specific ability to bind the identified cis-element in the *PbeGA2ox* promoter and activate its expression (**Figure 6**). While both are ABA-responsive bZIP transcription factors, they appear to have evolved distinct target gene specificities. In contrast, the expression of the ABA catabolic genes *PbeCYP707A-1* to *PbeCYP707A-5* were also significantly upregulated in the coatless embryos both treated with exogenous ABA and dormant seed coats (**Figure 3**). Considering the similar feedback regulation in *Prunus persica* and Arabidopsis (Wang and Deng, 2014; Shu et al., 2016a), we hypothesized that the upregulation could be due to feedback regulation by the higher ABA content in these embryos. Notably, although gene expression analysis revealed potential modulation of ABA/GA homeostasis during dormancy release, we emphasize that the absence of ABA and GA quantification constitutes a limitation. While established models indicate *NCED* upregulation typically elevates ABA levels and *GA2ox* induction promotes GA catabolism (Hedden, 2020; Aloryi et al., 2025), future direct hormone measurements will allow us to rule out potential decoupling between gene expression patterns and actual hormone concentrations caused by post-transcriptional regulation or compensatory metabolic pathways. The relative contributions of embryonic versus seed coat-derived ABA also deserves careful consideration. While qRT-PCR data showed rapid upregulation of *de novo* ABA biosynthesis genes in isolated embryos (**Figure 3**), potential priming effects from coat-translocated ABA prior to tissue isolation cannot be excluded. Future research will prioritize direct quantification of ABA levels to definitively resolve the contributions of seed coat-derived versus embryo-synthesized ABA in this system.

### 3.3 Molecular crosstalk between ABA and GA in regulating *Pyrus betulaefolia* seed dormancy

While studies have established the antagonistic relationship between abscisic acid and gibberellins in seed dormancy and germination (Lee et al., 2015; Hoang et al., 2014), the precise molecular mechanisms underlying their interaction remain incompletely understood. Crucially, seed coat-specific adaptations diverge across species: in Arabidopsis, endosperm-derived ABA transporters regulate embryonic ABI5 activation (Shimizu et al., 2022); in legumes, ABI5 directly promotes seed coat sclerification by repressing GA catabolism genes (Zinsmeister et al., 2016). AtABI4 has been identified as a crucial transcription factor in post-germination stages, which transcription and protein stability were enhanced by ABA, subsequently upregulating *AtGA2ox7* expression to reduce active GA content; Conversely, GA signaling suppresses AtABI4 expression and accelerates its protein degradation, thereby inhibiting *AtNCED6* transcription and ABA biosynthesis (Shu et al., 2016b). Cantoro et al. identified direct binding of *SbABI4* and *SbABI5* to the *SbGA2ox3* promoter *in vitro* using electrophoretic mobility shift assay, suggesting these transcription factors may trigger GA catabolism to suppress germination in dormant grains (Cantoro et al., 2013).

**Figure 7:**
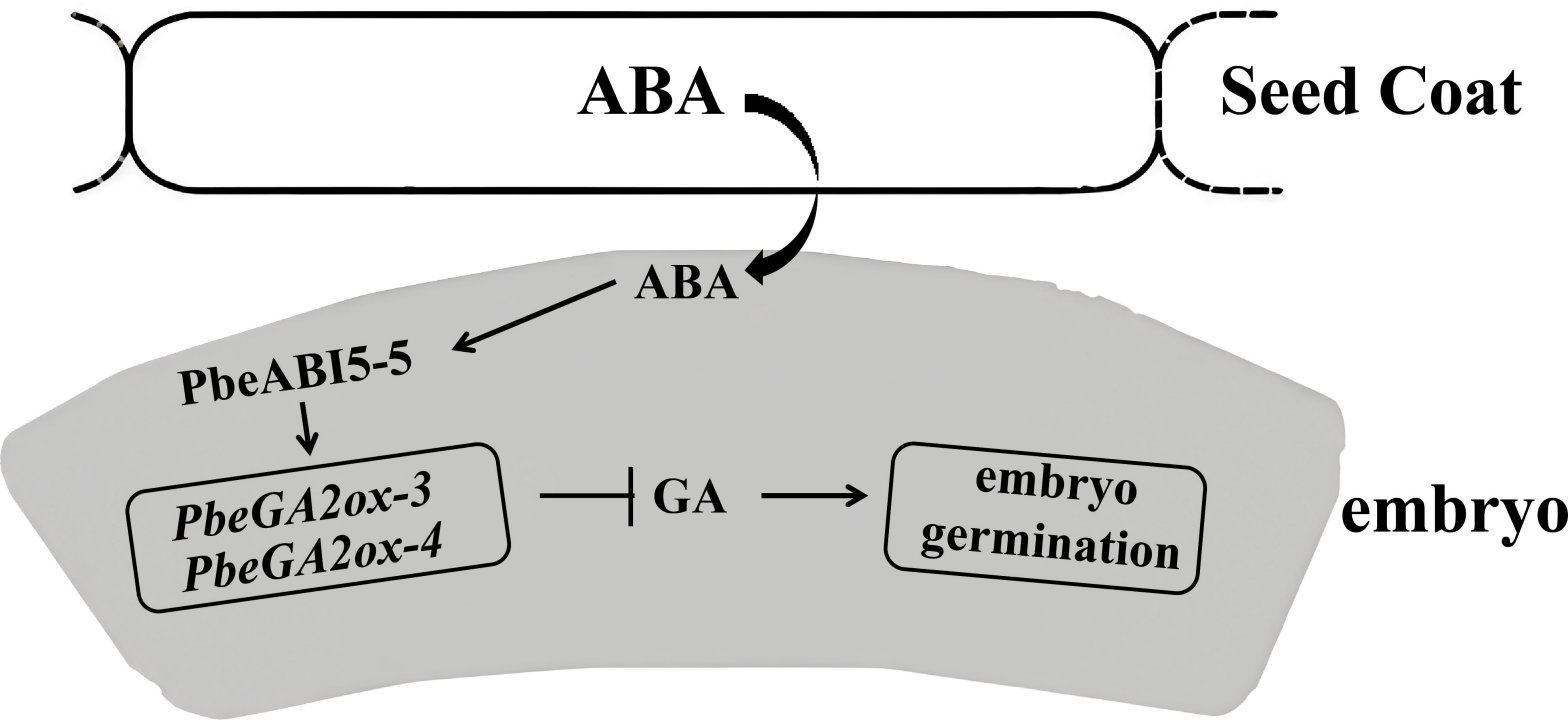
Proposed working model of seed coat- and ABA-dependent repression of dormant seed germination. Dormancy is a state where constitutive production of ABA in the coats maintains a proper level of PbeABI5 and repress GA biogenesis by the direct activation of *PbeGA2ox-3/4*, and therefore negatively regulates embryo germination, maintains dormancy. The lines ending with arrows denote positive regulation, while the lines ending with bars denote negative regulation. Note: GA promotes germination when present.

Our research reveals another key player in this hormonal crosstalk - PbeABI5-5. Based on the induction of *PbeGA2ox-3* and *PbeGA2ox-4* genes expression by ABA (**Figure 4**), we demonstrated that PbeABI5-5 directly bound to and activates the promoters of the two genes (**Figure 5**). Through yeast one-hybrid and tobacco transient expression assays, we demonstrate that PbeABI5-5 directly binds to and activates the promoters of *PbeGA2ox-3* and *PbeGA2ox-4* genes (**Figure 6**). These findings support our proposed model (**Figure 7**), where in *Pyrus betulaefolia* seed coats enhance embryonic ABA biosynthesis. Elevated ABA levels upregulate *PbeABI5-5* expression, which in turn activates *PbeGA2ox* genes through direct promoter binding. The resulting increase in GA catabolism reduces bioactive GA levels, maintaining embryonic dormancy by inhibiting germination. This regulatory cascade represents a novel mechanism through which ABA-dominated seed coat signaling influences embryonic GA metabolism to sustain dormancy.In conclusion, our study establishes that seed coat-derived ABA sustains dormancy in *Pyrus betulaefolia* by activating PbeABI5-5, which transcriptionally upregulates *PbeGA2ox* genes to enhance GA catabolism and suppress embryonic germination. This ABA-GA crosstalk mechanism, mediated through direct PbeABI5-GA2ox promoter binding, reveals a novel regulatory layer wherein seed coat signaling controls embryonic GA metabolism to maintain physiological dormancy.

## 4 Materials and methods

### 4.1 Plant materials and growth conditions

Freshly harvested *Pyrus betulaefolia* seeds were collected from Xinjiang Uygur Autonomous Region, China. The seeds were rinsed with sterile water and air-dried in a shaded environment before storage in self-sealing bags at room temperature for subsequent experiments.

For germination assay, seeds were surface-sterilized with 5% NaClO for 5 min, followed by five washes with sterile water. After a 4-h imbibition in sterile water, seeds were placed on two layers of moist filter paper in Petri dishes. Each dish was treated with 2 mL of sterile water (control), ABA, fluridone, GA, or PAC, depending on the experimental conditions. The plates were incubated in a growth chamber (22 ± 2°C, 60–70% relative humidity, 16-h light/8-h dark cycle). Germination was monitored twice daily, and sterile water or treatment solutions were replenished as needed to maintain moisture. Seeds with a 2-mm radicle protrusion were scored as germinated. The germination rate was calculated as: Germination rate (%) = (Number of germinated seeds/Total seeds) × 100.

For cold stratification, sterilized seeds were mixed with moist sand and stored at 4°C. After stratification, seeds were transferred to germination conditions as described above.

The seed coat bedding assay was adapted from Lee et al (Lee et al., 2010). Briefly, imbibed seeds were dissected into embryos and seed coats using fine forceps. Ten coatless embryos were placed on filter paper overlying 30 seed coats, supplemented with 2 mL of sterile water, ABA, or fluridone, and incubated under standard conditions (**Supplementary Figure 3**).

Water imbibition rates were determined by weighing seeds before and after incubation. Surface moisture was removed prior to initial weighing. The imbibition rate was calculated as: Water imbibition rate (%) = [(Post-incubation weight − Initial weight)/Initial weight] × 100.

### 4.2 RNA extraction and qRT-PCR analysis

The reference genome used was Pyrus x bretschneideri genome sequence. Total RNA was extracted using the HiPure Plant RNA Mini Kit (Magen, China) according to the manufacturer’s instructions. First-strand cDNA was synthesized from 2 µg of total RNA using the PrimeScript™ RT Reagent Kit with gDNA Eraser (Takara, Japan) following the manufacturer’s protocol. Quantitative real-time PCR (qRT-PCR) was performed using SYBR Premix Ex Taq™ (Takara, Japan), on a CFX96 Real-Time PCR System (Bio-Rad, USA). Gene-specific primers (designed with Primer3, http://bioinfo.ut.ee/primer3-0.4.0/) are listed in **Supplementary Table S1**. Prior to analysis, primer efficiency was validated using a standard curve (80–120% efficiency required). Each reaction was run in three technical replicates, and gene expression levels were calculated using the 2^-ΔΔCt^ method, with *PpActin* (JN684184) as the internal reference (Liu et al., 2012).

### 4.3 ABRE motifs analysis of *PbeGA2ox* promoters

The 2000bp region upstream from the start codon of *PbeGA2ox* genes were extracted from the *Pyrus betulaefolia* genome as a promoter. Then, the sequences of promoters were uploaded to the Plantcare (http://bioinformatics.psb.ugent.be/webtools/plantcare/html/) to predict ABRE motifs.

### 4.4 Yeast one-hybrid assay

Yeast one-hybrid (Y1H) assays were performed using the Matchmaker™ Gold Yeast One-Hybrid Library Screening System (Clontech, USA) following the manufacturer’s protocol. The primers used for constructing bait and prey are listed in **Supplementary Table S2**. Different promoter fragments of *PbeGA2ox* containing the ABRE sequence were cloned into the pAbAi vector. The recombinant plasmid was linearized and integrated into the genome of the Y1H Gold yeast strain. Subsequently, yeast cells were co-transformed with either PbeABI5-AD or the empty AD vector. Protein-DNA interactions were assessed based on yeast growth on SD/-Leu medium supplemented with 100–200 ng/mL aureobasidin A.

### 4.5 Dual-luciferase reporter assay

The dual-luciferase assay was performed in *Nicotiana benthamiana* leaves following the method of Niu et al (Niu et al., 2016). The full-length coding sequences of *PbeABI5-1* and *PbeABI5-5* were cloned into the pGreenII 0029 62-SK vector, while the promoter regions of *PbeGA2ox-3* and *PbeGA2ox-4* were inserted into pGreenII 0800-LUC. All constructs were transformed into *Agrobacterium tumefaciens* strain GV3101 using the freeze-thaw method. For infiltration, *Agrobacterium* cultures (OD_600_ = 1.0) carrying the effector and reporter constructs were mixed at a 10:1 ratio and incubated at room temperature for 2 h before infiltration into leaves. After 48-72 h post-infiltration, leaf samples were collected for observation. For qualitative detection, luciferase activity was visualized using the NightSHADE LB 985 imaging system (Berthold Technologies) after infiltration of 0.2 mM luciferin (Promega) 30 min prior to imaging. For quantitative analysis, firefly and Renilla luciferase activities were measured using the Dual-Luciferase^®^ Reporter Assay System (Promega) on a Modulus™ Luminometer (Promega). Reporter gene expression was calculated as the ratio of LUC_firefly_: REN_Renilla_. Each experiment included three independent biological replicates, with six technical replicates per biological replicate.

### 4.6 Statistical analysis

All experiments were arranged in a completely randomized design. Data were analyzed with SPSS Statistics v.18.0 (IBM, USA). Statistical significance was determined at *p < 0.05* using appropriate tests. Data visualization was performed using GraphPad Prism 7.00. Values are presented as mean ± standard error (SE) from at least three independent biological replicates.

## Data Availability

The original contributions presented in the study are included in the article/**Supplementary Material**. Further inquiries can be directed to the corresponding authors.
