# Supplementary material for: Seed coat-derived ABA regulates seed dormancy of Pyrus betulaefolia by modulating ABA and GA balance
Source: Front Plant Sci. 2025 Sep 1;16:1667946. doi: 10.3389/fpls.2025.1667946 (PMC12414344; doi:10.3389/fpls.2025.1667946)
Supplement: Supplementary file 1 [file DataSheet1.pdf]

Supplementary Material

1 Supplementary Figures and Tables

1.1 Supplementary Figures

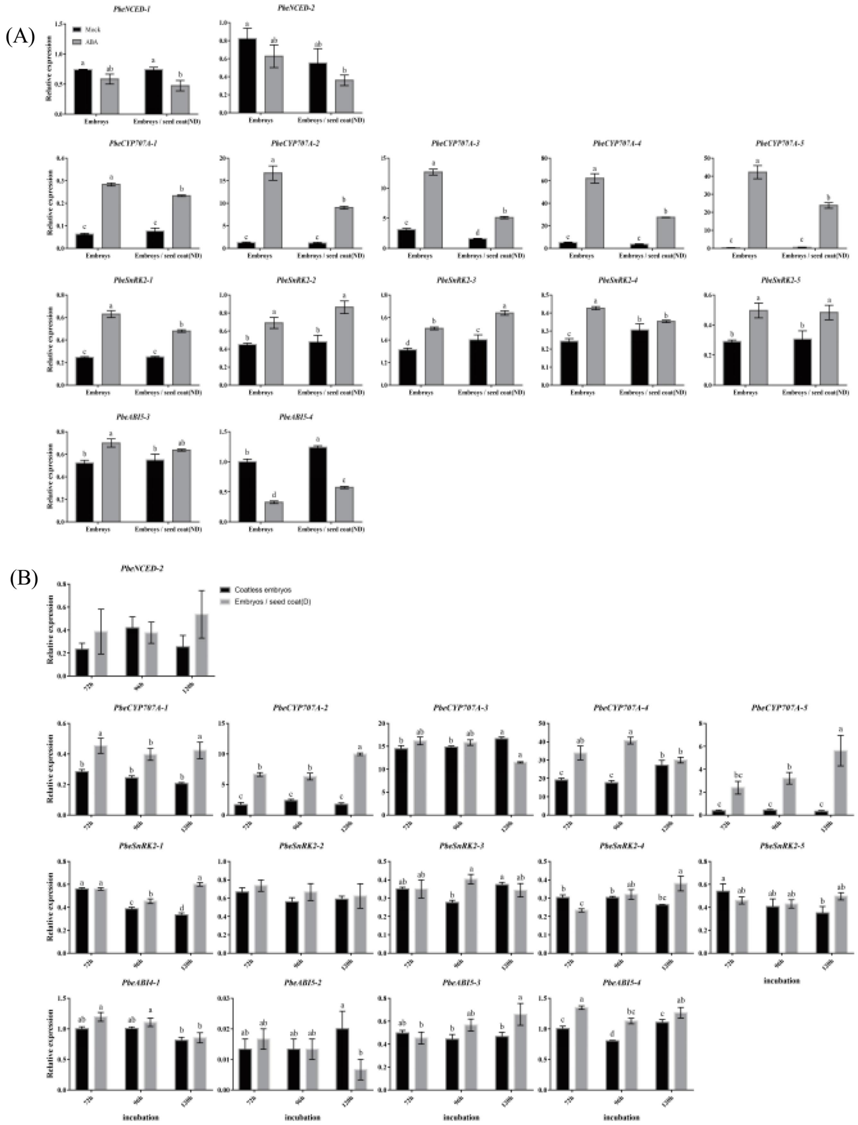

Supplemental Figure 1. Expression profiles of ABA related genes at different conditions. (A) Total RNA was extracted from coatless embryos and the embryos co-incubated with nondormant seed coats sampled after 120 h of incubation in the absence or presence of 100 µM ABA. **(B)** Total RNA was extracted from coatless embryos and the embryos co-incubated with dormant seed coats sampled at 72, 96, and 120 h after the beginning of incubation. Relative expression levels of ABA-related genes were determined by qRT-PCR as described in the Materials and Methods. The bars represent the mean ± SEM of three biological repeats. Different letters indicate a significant difference among sampling time points ($p < 0.05$).

(A)

(B)

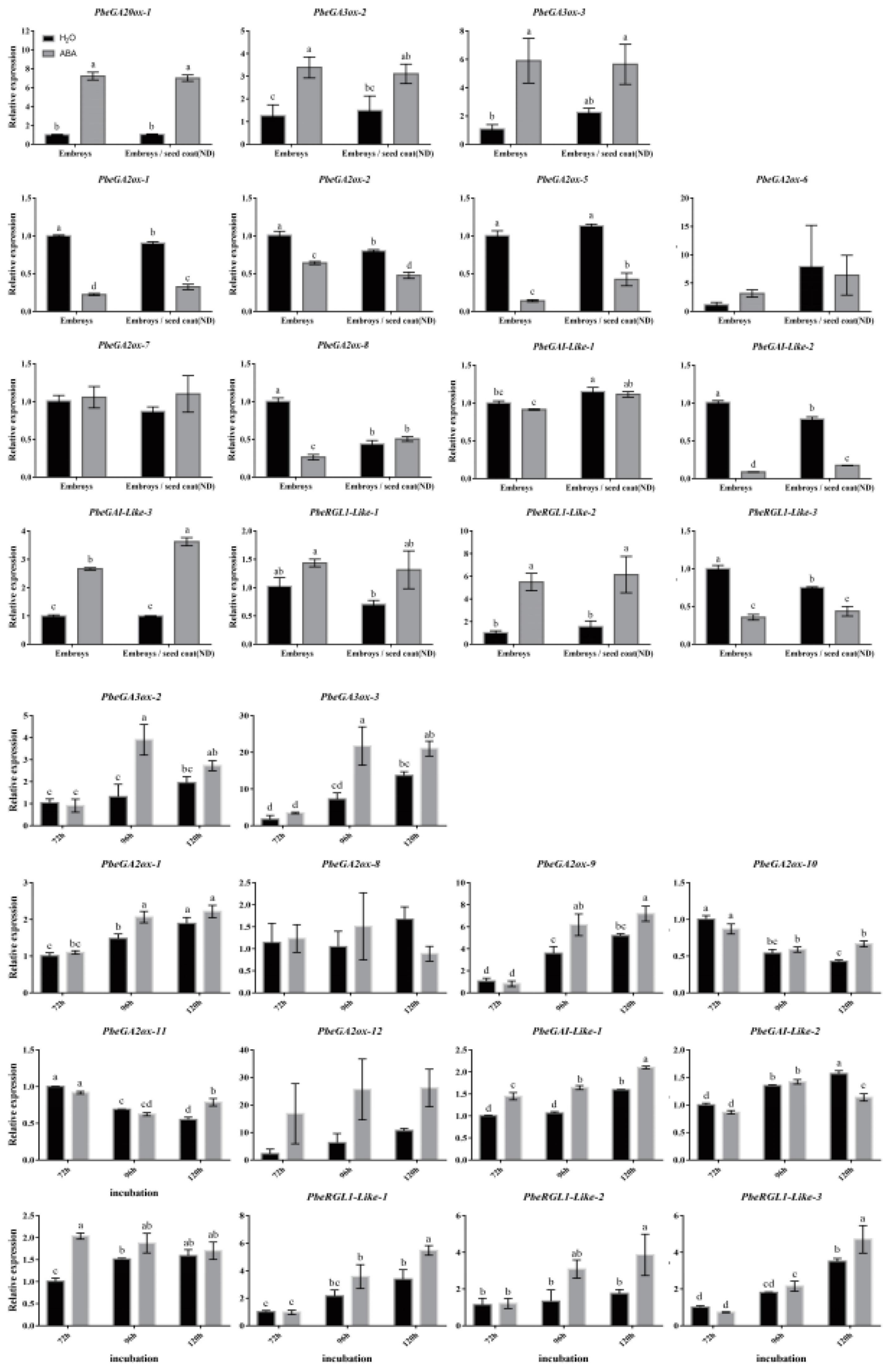

Supplemental Figure 2. Expression profiles of GA related genes at different conditions. (A) Total RNA was extracted from coatless embryos and the embryos co-incubated with nondormant seed coats sampled after 120 h of incubation in the absence or presence of 100 μM ABA. **(B)** Total RNA was extracted from coatless embryos and the embryos co-incubated with dormant seed coats sampled at 72, 96, and 120 h after the beginning of incubation. Relative expression levels of GA-related genes were determined by qRT-PCR as described in the Materials and Methods. The bars represent the mean ± SEM of three biological repeats. Different letters indicate a significant difference among sampling time points ($p < 0.05$).

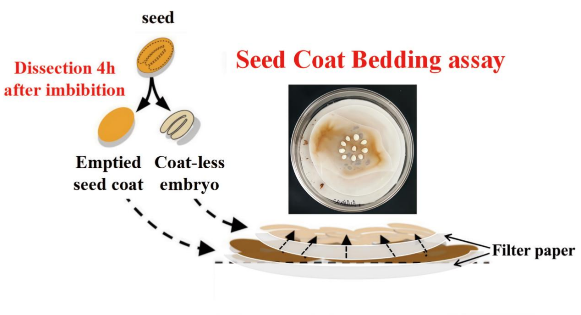

Supplemental Figure 3. Embryos and seed coats are dissected 4 h after seed imbibition and coatless embryos are laid on a layer of dissected seed coats.

1.2 Supplementary tables

Supplemental Table 1. Primers used for qRT PCR analysis

mRNA	Gene	Primer-F (5'-3')	Primer-R (5'-3')
PbeNCED-1	Pbr025271.1	CTACAAGACACCGCCACCTT	AGTGGGAGTTGAAGGTTGTTGA
PbeNCED-2	Pbr039596.1	AAGGAAGCCACTCGAGCAAA	TTTGGACACGTGGGTCTGAG
PbeNCED-3	Pbr006012.1	CATGGACCCACCGGATTCAA	AGCAATACCCGAACACCTGG
PbeSnRK2-1	Pbr003186.1	TGAAATACATCGAACGCGGC	TCCTCTCAAAGAGCTCTCCAC
PbeSnRK2-2	Pbr040625.1	GGCGAATCCTTACTGTACGCT	TCTATGCTCTGGGCTGGAGT
PbeSnRK2-3	Pbr042784.1	CCAGAGTGCCGCCATCTAAT	GGCTTTGCATGGGTTGATCC
PbeSnRK2-4	Pbr040276.1	TCCCAGTACACCATACCCGA	TGTTTAGGCTGGTCACGCTC
PbeSnRK2-5	Pbr026536.1	TGTTGCAAATCCAAGGATCACC	ATCGGGAAACTGGAGGAGGA
PbeABI3-1	XM_009374173.1	TTGGGACTTCTCGTGTGTGG	TGCCGTACTTTCACTCCTCG
PbeABI4-1	XM_009347499.1	ACCAACGACAAGTACCCCAC	AATATCCGAACAGCCCGGTG
PbeABI5-1	XM_009376454.1	GAGGAGGCAGAGAAGGATGA	TCTCCTTCACTTTCTGGGCC
PbeABI5-2	XM_009366032.1	TTGCCAGAATCGAGTAGCGG	ATACCTCTTGCTTGCGCCTT
PbeABI5-3	XM_009340957.1	CCAACCTATACCACCGCCAA	CCCAGGTGTCTGCGTATCTG
PbeABI5-4	XM_009347981.1	GGAATTGGCACGGTTGGTTT	GGTACCGGCGACAATGAAGA
PbeABI5-5	XM_009361373.1	TTAATGGCGGCTTTAGGGGG	TCTTCCCGCGTTGAAGGTTT
PbeCYP707A-1	Pbr004630.1	CTCTTGGCAAGGAAGAGGCT	AGCTGTAGTGTCTTGGGCTG

PbeCYP707A-2	Pbr019636.1	TCAGCTCCCTCCAGGTTCAT	GGGATACGTGGGCTTGAACA
PbeCYP707A-3	Pbr029414.1	GATGCCCATGACTTGTAGGGTT	CGCTGCCAAATGGCATGTAT
PbeCYP707A-4	Pbr006776.1	AGCCAAGCTGGAGATTTTGGT	CTGATGGGCAGGCCATTTTG
PbeCYP707A-5	Pbr003860.1	TGCATTCTTGTCCAGGCAGT	GCTTGGGTACTGGGAAAGGG
PbeGA20ox-1	XM_009372977.2	GGTGATGACTAATGGGCGGT	GCCATTAGAGACGGCAGAGG
PbeGA20ox-2	XM_009381346.1	CACAGGGTTTTGGCAAATGGT	TAGGCTTTCCTCCTCTCCGTT
PbeGA3ox-1	XM_009345150.1	AGTGAACCGGACCCAACATC	GGAGCACAAAGTCTGACCGA
PbeGA3ox-2	XM_009356768.1	CTACCGTTGGCTCACCACTT	TGAGCCAAGCATTAGCCACA
PbeGA3ox-3	XM_009356874.1	TGTCCATTGTCCACCAGAGC	GTTGTGCCCGTTTCACCAAG
PbeGA2ox-1	XM_009362611.1	TGGTGATTGCTTGCAGGTGA	TCAAAGGTGGCCCTCCAAAG
PbeGA2ox-2	XM_009356580.1	ACGGAGAGGAGGAAAGCCTAT	ATTGGAGGAAGGGGCAAAACA
PbeGA2ox-3	XM_009373809.1	TGTTGCAGGCCATGACAAATG	TCCGGTGTGACCATTTCTGG
PbeGA2ox-4	XM_009369005.1	GCCCAGCCTCTACAAACCTT	TCGTTGCACTTGCAGGTATGA
PbeGA2ox-5	XM_009340651.1	CATGATGCAGGCGATAAGCG	CTCCGAATCACACTGCCTTCT
PbeGA2ox-6	XM_009342659.1	CGAATGAACCGATATCCGCCT	CTGCGATCCATCTGTCGTCT
PbeGA2ox-7	XM_009342783.1	CACAAGGTGATGGCAAACGAG	AGGAATCTCGAAAGGCCGAC
PbeGA2ox-8	XM_009346791.1	GGTGTTACCGCAGGTGGATT	GGAAGAGTCACCAACAGCGA
PbeGA2ox-9	XM_009346923.1	CGCAAGTGTTTGGCCTCATC	GGCCTCAAAGAAGTCCCCAA
PbeGA2ox-10	XM_018646813.1	TCCCGGCACTTGCTATCTTC	TGATCAACTGCAATCCGCCA
PbeGA2ox-11	XM_009369486.1	AAGTGTGAGGCACAGGGTTT	CAAGTCTTGAGCTGTAGGCAGA
PbeGA2ox-12	XM_009380615.1	CGAGTGGTTACCGGACAAGAA	ATGGTTCATCACCGCAGCTT
PbeRGL1-Like-1	XM_009348352.1	GAGGGGAGCGAAAGGGTTAC	GGAGTTCCCTTCCACCCAAC
PbeRGL1-Like-2	XM_009371729.1	CTCTCAATTCGCGCTGAGGA	GGCGAGTTGTGATTTGCCTC
PbeRGL1-Like-3	XM_009380478.1	GTGAGAAGCGTGACGATGGA	ATTGGGGTTCCTTTCCACCC

PbeGAI-Like-1	XM_009351239.1	GCGAACAGTCGGGATAAGGT	CGAGATGAACCGGGACGAAA
PbeGAI-Like-2	XM_009373135.1	CCTGGATCCGGAACCTCAAC	AGTAGTACAGCGCTTCCGTG
PbeGAI-Like-3	XM_009336173.1	GGGTGGAGGAGAACAATGGG	GCCCAGTGGTGCAATCAATC

Supplemental Table 2. Promoter and full-length TFs amplification primers for yeast one-hybrid assay

Gene	Primer (5'-3')
ProGA2ox3-F-P1	GAAAAGCTTGAATTCGAGCTCACAATTAATTAAATTCCTAATC
ProGA2ox3-R-P1	AGCACATGCCTCGAGGTCGACTAATGAACCATTGATTTATTTG
ProGA2ox3-F-P2	GAAAAGCTTGAATTCGAGCTCCAAATTTGTCGATTGTAATCAC
ProGA2ox3-R-P2	AGCACATGCCTCGAGGTCGACTGGTCCATATTTGTGGTTCACC
ProGA2ox3-F-P3	GAAAAGCTTGAATTCGAGCTCATGTTGAGTTTGATGATCATTG
ProGA2ox3-R-P3	AGCACATGCCTCGAGGTCGACATTGCTGACTATTTATCGATGC
ProGA2ox3-F-P5	GAAAAGCTTGAATTCGAGCTCGTGCATGCTATACGTTCTCGTG
ProGA2ox3-R-P5	AGCACATGCCTCGAGGTCGACAACAGTATAAGTACCAAGTAAC
ProGA2ox4-F-P1	GAAAAGCTTGAATTCGAGCTCAGAAGACAAGAGGTGCACGTGTG
ProGA2ox4-R-P1	AGCACATGCCTCGAGGTCGACCACCAAGTGTCATAATACAAATGG
ProGA2ox4-F-P2	GAAAAGCTTGAATTCGAGCTCTTTGAAAATTTCAAAGTCTCATGG
ProGA2ox4-R-P2	AGCACATGCCTCGAGGTCGACACGCGTATATATGTTTCTTTGATG
ProGA2ox4-F-P3	GAAAAGCTTGAATTCGAGCTCTTTTCAGTGTACCGAACATG
ProGA2ox4-R-P3	AGCACATGCCTCGAGGTCGACTTCAAATATCCAAACACTTG
PbeABI5-1-AD-F	GCCATGGAGGCCAGTGAATTCATGGGTGTTTCAGAGTCGGAAATC
PbeABI5-1-AD-R	CAGCTCGAGCTCGATGGATCCTCACAAACCACAACTATGACTCCT
PbeABI5-5-AD-F	GCCATGGAGGCCAGTGAATTCATGGGGACCAACATGAACTTCAAG
PbeABI5-5-AD-R	CAGCTCGAGCTCGATGGATCCTCACCACGGACCCGTCATCGTTCT

Supplemental Table 3. Promoter and full-length TFs amplification primers for dual-luciferase reporter assay

Gene	Primer (5'-3')
ProGA2ox3-LUC-F	TCGAGGTCGACGGTATCGATAACAATTAATTAAATTCCTAATC
ProGA2ox3-LUC-R	CCGCTCTAGAACTAGTGGATCTAATCTAAGAGATGTGGTGC
ProGA2ox4-LUC-F	TCGAGGTCGACGGTATCGATAAGAAGACAAGAGGTGCACGTGTG
ProGA2ox4-LUC-R	CCGCTCTAGAACTAGTGGATCAAGGAGTAAGATCTAGGATAAG
PbeABI5.1-SK-F	GCGGCCGCTCTAGAACTAGTGATGGGTGTTTCAGAGTCGGAAATC
PbeABI5.1-SK-R	GGTCGACGGTATCGATAAGCTTCACAAACCACAACTATGACTCCT
PbeABI5.5-SK-F	GCGGCCGCTCTAGAACTAGTGATGGGGACCAACATGAACTTCAAG
PbeABI5.5-SK-R	GGTCGACGGTATCGATAAGCTTCACCACGGACCCGTCATCGTTCT
